# Dietary intake during a pragmatic cluster-randomized weight loss trial in an underserved population in primary care

**DOI:** 10.1186/s12937-023-00864-7

**Published:** 2023-08-02

**Authors:** John W. Apolzan, Corby K. Martin, Robert L. Newton, Candice A. Myers, Connie L. Arnold, Terry C. Davis, William D. Johnson, Dachuan Zhang, Christoph Höchsmann, Vivian A. Fonseca, Kara D. Denstel, Emily F. Mire, Benjamin F. Springgate, Carl J. Lavie, Peter T. Katzmarzyk, PROPEL Research Group, Phillip Brantley, Ronald Horswell, Tina K. Thethi, Jonathan Gugel, Eboni Price-Haywood, Kathleen B. Kennedy, Daniel F. Sarpong

**Affiliations:** 1 Pennington Biomedical Research Center, Louisiana State University System, 6400 Perkins Road, Baton Rouge, LA USA; 2 Department of Medicine, Feist-Weiller Cancer Center, Louisiana State University Health Sciences Center, Shreveport, LA USA; 3 Department of Sport and Health Sciences, Technical University of Munich, Munich, Germany; 4 Department of Medicine, Division of Endocrinology and Metabolism, Tulane University Health Sciences Center, School of Medicine, Southeast Louisiana Veterans Health Care System, New Orleans, LA USA; 5 Department of Internal Medicine, Section of Community and Population Medicine, Louisiana State University School of Medicine, New Orleans, LA USA; 6 Program in Health Policy and Systems Management, School of Public Health, Louisiana State University, New Orleans, LA USA; 7 Department of Cardiovascular Diseases, John Ochsner Heart and Vascular Institute, Ochsner Clinical School-The University of Queensland School of Medicine, New Orleans, LA USA

**Keywords:** Lifestyle Intervention, Energy Restriction, Weight loss, Diet, Age, Race, Sex, Food Insecurity, Underserved

## Abstract

**Background:**

Currently there are limited data as to whether dietary intake can be improved during pragmatic weight loss interventions in primary care in underserved individuals.

**Methods:**

Patients with obesity were recruited into the PROPEL trial, which randomized 18 clinics to either an intensive lifestyle intervention (ILI) or usual care (UC). At baseline and months 6, 12, and 24, fruit and vegetable (F/V) intake and fat intake was determined. Outcomes were analyzed by repeated-measures linear mixed-effects multilevel models and regression models, which included random cluster (clinic) effects. Secondary analyses examined the effects of race, sex, age, and food security status.

**Results:**

A total of 803 patients were recruited. 84.4% were female, 67.2% African American, 26.1% received Medicaid, and 65.5% made less than $40,000. No differences in F/V intake were seen between the ILI and UC groups at months 6, 12, or 24. The ILI group reduced percent fat at months 6, 12, and 24 compared to UC. Change in F/V intake was negatively correlated with weight change at month 6 whereas change in fat intake was positively associated with weight change at months 6, 12, and 24 for the ILI group.

**Conclusions:**

The pragmatic weight loss intervention in primary care did not increase F/V intake but did reduce fat intake in an underserved population with obesity. F/V intake was negatively associated with weight loss at month 6 whereas percent fat was positively correlated with weight loss throughout the intervention. Future efforts better targeting both increasing F/V intake and reducing fat intake may promote greater weight loss in similar populations.

**Trial registration:**

NCT Registration: NCT02561221

**Supplementary Information:**

The online version contains supplementary material available at 10.1186/s12937-023-00864-7.

## Introduction

The public health consequences of obesity and its associated diseases are immense. In the United States, the current obesity prevalence among adults is 42% [1], and ~ 2/3 of adults have overweight or obesity [2]. A first line treatment for obesity is lifestyle intervention. However, there are limited pragmatic lifestyle interventions, particularly those designed for underserved populations, to test if these approaches may be valuable for improving aspects of dietary intake during weight loss.

The U.S. Department of Agriculture (USDA) continues to promote a lifestyle in which the diet is high in fruits and vegetables and has modest fat intake. The Acceptable Macronutrient Distribution Range (AMDR) for dietary fat is currently 20–35% and 2.0 cups of fruits and 2.5 cups of vegetables are recommended (about 4.5 daily servings) [3]. The average American is near the upper recommendation for fat intake and, unfortunately, the majority of Americans do not meet the targets for fruit (13.1%) and vegetable (8.9%) intake [4].

Improvements in dietary intake are well established during weight loss trials conducted in academic health centers [5–9]. For example, The Diabetes Prevention Program (DPP) and the Weight Loss Maintenance (WLM) trials demonstrated increased fruit and vegetable intake and decreased fat intake [6, 8, 9]. Through these trials it is well known that improvements in diet yield better long-term weight loss [7, 8]. However, well-powered weight loss trials in primary care are limited. For example, the POWER-UP trial, conducted in 6 primary care clinics, did not find differences in dietary intake over 24 months of intervention [10], unlike the previous trials conducted within academic health centers.

The Promoting Successful Weight Loss in Primary Care in Louisiana (PROPEL) trial offers a unique opportunity to examine changes in dietary intake during weight loss in primary care. The primary findings have previously been published; the weight loss intervention in an underserved population was successful with about 4.5% more weight loss maintained at 2 years compared to usual care [11]. The study was powered to examine differences in weight loss across the heterogeneity of subgroups including race, sex, and age. Since dietary intake was a planned secondary outcome of the trial, this manuscript examines changes in fruit and vegetable intake and percent fat in the diet. Thus, the objectives of the present analyses closely resemble that of the primary trial. Since rigorous weight loss trials previously found improvements in diet, we hypothesized that the intensive lifestyle intervention (ILI) would increase fruit and vegetable intake and decrease percent fat intake compared to usual care (UC) in the PROPEL trial. Furthermore, we hypothesized that dietary improvement (s) would be associated with greater weight loss.

## Methods

### Patients

Patients were 20–75 years old, had a BMI in the range of 30 – 50 kg/m^2^, and were recruited from participating clinics. Full exclusion criteria are listed elsewhere [12] but included current participation in a structured weight loss program, use of weight loss medication, plans to move during the study duration, given birth within the past year, and past metabolic surgery.

### Study design

The trial design (including assessment procedures and intervention) and baseline subject characteristics have been published in detail [12, 13]. The PROPEL trial tested the effects of a 24-month ILI compared to UC, in primary care clinics across the state of Louisiana. The eighteen clinics were randomly allocated to either the ILI or UC group. The trial was conducted between April 2016 and September 2019.

The screening, baseline, month 6, 12, 18, and 24 visits were conducted at the primary care clinics. The diet and lifestyle questionnaires were not completed at the month 18 visit, thus data from this visit are not included in the current manuscript.

### Intervention

The UC group maintained their normal usual care through their primary care team for the duration of the 24-month study. To maintain contact, UC patients were provided three newsletters per year (6 total) on health-related topics.

Trained health coaches delivered the ILI as recommended by the 2013 AHA/ACC/TOS Obesity Guidelines [14]. The intervention was adapted for health literacy and based upon the interventions delivered in the DPP [15], Look AHEAD [16], and CALERIE [17] studies. The first 6 months included planned weekly contact with 16 in-person sessions and 6 phone calls. The following 18 months alternated between monthly face to face and phone sessions although patients who needed additional support were encouraged to have more frequent contact with their health coach which may have included text messages, phone calls, or in-person sessions. In all, there were 43 planned contacts between the health coach and patient over 2 years. The sessions have been previously published in the supplemental material [12], but over 50% of sessions focused on dietary intake changes. If participants were performing sub optimally, a ‘toolbox’ of strategies was utilized to help achieve ILI goals [15–17]. Some toolbox options included: 1) Weigh yourself everyday, 2) Keep track of what you are eating, 3) Your coach will contact every few days, 4) Swap some of your foods for healthier ones, 5) Write down how much activity you are doing, 6) Practice paying more attention to what, when, and how you feel when you eat, 7) Remove the foods and cues that make you want to eat more and move less.

A personalized goal of 10% weight loss over the first 6 months of the trial was set for each of the patients. During the first 4 weeks of the trial a meal plan was provided that included portion-controlled foods. This included pre-packaged portion-controlled food, meal replacement shakes, as well as other options including fruit and soup. During week 5, the meal plan became slightly less structured as the pre-packaged, portion-controlled foods and meal replacement shakes became a toolbox option. Overall, the meal plans were consistent with dietary guidelines with recommendations being ~ 55% carbohydrate, ~ 15% protein, and 30% fat. Furthermore, the servings of nutrient dense foods were recommended i.e., fruits, vegetables, whole grains, and lean protein. Health coaches attempted to work with patients to find available and affordable options for fruits and vegetables (F/V). These included in season F/V, frozen F/V, etc. Also, if warranted, health coaches tried to locate food pantries if patients were willing and in need of assistance.

Besides dietary quality, the intervention encouraged increased physical activity (~ 175 min week), as well as daily weighing using a cellular connected scale. Patients’ body weights were plotted daily on the computerized tracking system (CTS). The CTS housed a weight loss calculator which was used to calculate personalized energy intake targets for each participant to reach 10% weight loss and programmed a graph that displayed the patients anticipated weight loss over time. The weight graph included a zone which demonstrated if patients were adherent to their energy intake target. This zone determined if a patient was adherent to their energy intake prescription and losing and/or maintaining weight at the expected rate [18–21].

Anthropometrics:

Height was measured with a portable stadiometer (Seca Model 213) at the baseline visit to the nearest 0.1 cm. Body weight and waist circumference were measured at all study clinic visits with a with a digital scale (Seca Model 876) and a non-elastic anthropometric tape (Graham Field Model 1340–2), respectively. All measurements were performed in duplicate. If the two measurements differed by more than 0.5 cm, 0.5 kg, and 0.5 cm for height, weight, and waist circumference, respectively, a third measurement is obtained, and the two closest measurements were averaged for analysis.’

### Questionnaires

#### Baseline demographic and health history questionnaire

A self-report demographic and health history questionnaire asked patients their age, sex, race/ethnicity, health insurance status, income, employment, and education level.

#### Dietary intake questionnaires

Dietary fat, fruit, and vegetable intake were examined. Several questionnaires were combined into a single dietary screener. The National Cancer Institute (NCI) fat screener estimates the percentage of energy from fat by reporting the frequency of consuming specific foods over the past 12 months [22]. The standard 7-item fruit and vegetable (F/V) screener developed by the NCI and National 5 a Day Program asked how many servings of fruit and vegetables were consumed in the past month [23, 24].

#### Food security

Household food security was measured using a 6-item subscale of the 12-month Food Security Scale Questionnaire [25]. Two or more affirmative responses represented food insecurity versus food security.

## Statistical Analysis

Difference in change in F/V intake and percent fat between the ILI and UC group outcomes at 6, 12, and 24 months were analyzed in the context of repeated-measures linear mixed-effects multilevel models, which included random cluster (clinic) effects. In addition to trial group, assessment time, baseline, and their interaction terms, the models included age, sex, and race as covariates. The within-patient correlation was accounted by imposing covariance structure upon the random residuals term of patients. Covariance structures were chosen based on the lowest AIC. We performed intention-to-treat analyses, which included all patients (regardless of the number of assessments obtained) and used the restricted maximum-likelihood method. We also investigated the group differences within strata of age, sex, race, and food security status. The model assumed that missing values were missing at random, and all values presented in the tables and figure are model-based estimates. The model can simply be expressed as:

$$\Delta Dietary Intake=\alpha +{G}_{l}+{T}_{k}+G\times {T}_{lk}+{C}_{j}+{C\times T}_{jk}+Race+Sex+Age+{e}_{ijkl}$$

where G is group, T is time, C is clinic, and e is the unobserved vector of random errors having an unstructured covariance matrix.

In the subgroup analyses, we assessed the relationship between outcome variables (weight change and waist circumference change) and change in dietary (F/V and fat) intake variables using mixed-effects regression models. Stratifying by four factors, namely, race, sex, age category, and food security status, we performed a separate regression model for each subgroup at each time point for ILI group only. The model could be described as:

$${Weight Loss}_{ij}={\beta }_{0}+{\beta }_{1}{\Delta Dietary Intake}_{ij}+{\delta }_{j}+{\varepsilon }_{ij}$$

At a particular time, ${Weight Loss}_{ij}$ represents follow-up minus baseline weight of the $ith$ patient $(i=\mathrm{1,2},\dots ,{n}_{j})$ in clinic $j (j=\mathrm{1,2},\dots ,m)$ and $m$ represents the number of clinics within the specific subgroup. The regression model incorporated cluster randomization by including a random effect term for clinic-specific intercept denoted as ${\delta }_{j}$. ${\varvec{\delta}}$ follows a multivariate normal distribution with mean as m-dimensional null vector, and covariance as ${n}_{j}\times {n}_{j}$ block diagonal matrix with $m$ blocks of ${\sigma }_{c}^{2}$ (variance component of the vector of random cluster effect) on the diagonal. ${\varepsilon }_{ij}$ denotes the experimental unit error term and ${\varepsilon }_{ij}\sim N\left(0,{\sigma }^{2}\right)$. To address the possible nonlinearity in the relationship, ILI participants were divided into tertiles based on ranks of dietary intake change at a specific time point and mixed model ANOVA was run with weight loss as dependent variables. All analyses were conducted with SAS version 9.4 (SAS Institute Inc., Cary, NC) for Windows with the significance level set to 0.05 (2-sided) and presented as Mean ± Standard Error. Tables are presented as 95% Confidence Intervals (95% CI).

## Results

The CONSORT diagram and baseline characteristics of the sample have previously been published [11, 12, 26]. Briefly, 803 adult patients (452 ILI and 351 UC) which were 67% African American (AA) and 84% female were recruited. The average age of the sample was 49.4 years and BMI of 37.2 kg/m^2^.

The overall baseline F/V intake was 2.2 ± 0.1 servings / day and percent fat was 35.3 ± 0.2% (p˃0.05). Similarly, the ILI had 2.2 ± 0.1 F/V servings a day and 35.9 ± 0.3% energy from fat while usual care had 2.3 ± 0.1 F/V servings a day and 34.6 ± 0.3% energy from fat (p˃0.05).

**Fig. 1:**
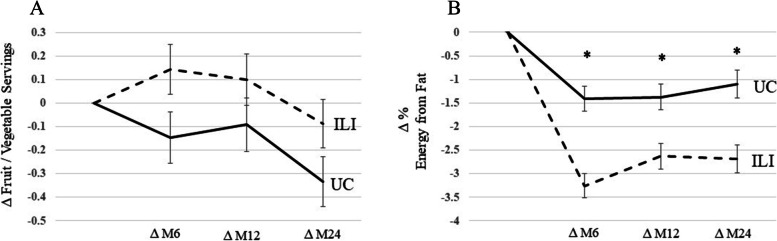
Change in Fruit / Vegetable Intake and % Energy from Fat during the PROPEL trial. 1A is change in fruit / vegetable servings during the PROPEL trial. 1B is change in percent fat intake during the PROPEL trial. * denotes *p* < 0.05

No differences in change in F/V intake were seen between the ILI and UC groups during the course of the intervention (Fig. 1A). However, the ILI group reduced percent fat intake at months 6, 12, and 24 compared to UC (Fig. 1B).

**Fig. 2:**
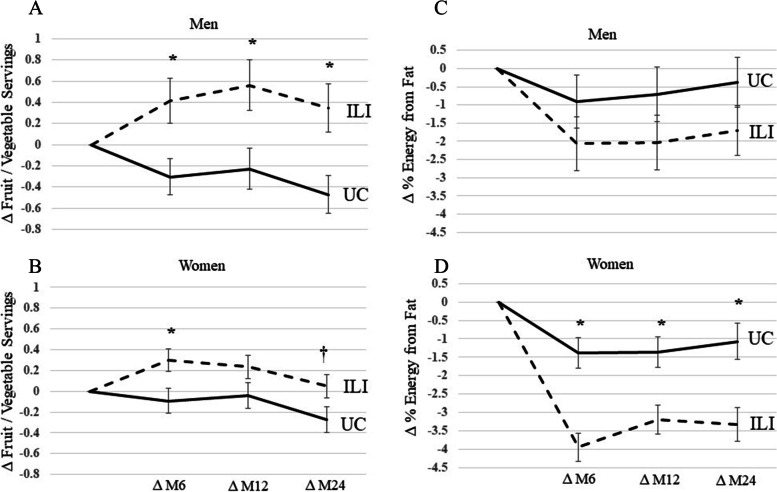
Change in Fruit / Vegetable Intake and % Energy from Fat by Sex during the PROPEL trial. 2A and 2C are change in fruit / vegetable servings and percent fat intake in men during the PROPEL trial. 2B and 2D are change in fruit / vegetable servings and percent fat intake in women during the PROPEL trial. * denotes *p* < 0.05, † denote *p* < 0.07

We examined changes in F/V intake and percent fat by sex. Men in the ILI increased F/V intake at months 6, 12, and 24 compared to men in UC (Fig. 2A). However, women in ILI only increased F/V intake at month 6 and tended to increase at month 24 compared to UC (Fig. 2B). No differences were seen between groups at month 12 among women. Men in ILI did not reduce percent fat compared to UC at any time point (Fig. 2C). However, women in ILI reduced percent fat at months 6, 12, and 24 compared to UC (Fig. 2D).

**Fig. 3:**
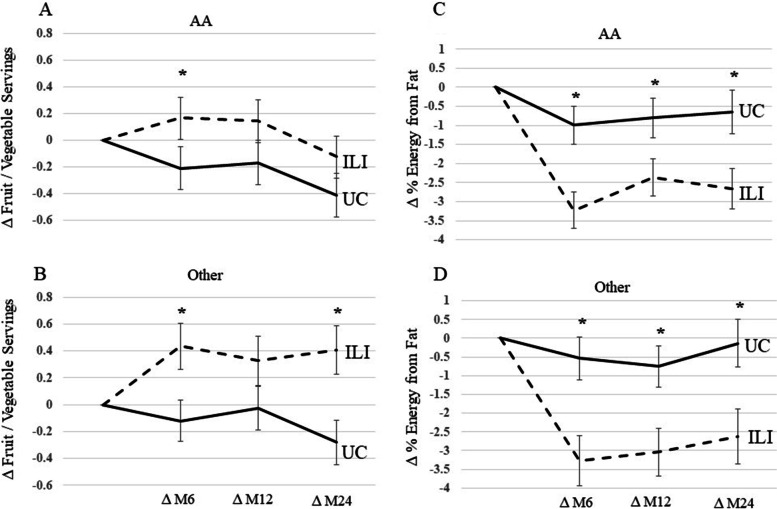
Change in Fruit / Vegetable Intake and % Energy from Fat by Race during the PROPEL trial. 2A and 2C are change in fruit / vegetable servings and percent fat intake in AA during the PROPEL trial. 2B and 2D are change in fruit / vegetable servings and percent fat intake in other races during the PROPEL trial. * denotes *p* < 0.05, † denote *p* < 0.07

Next, we examined change in F/V intake and percent fat by race. AA in the ILI increased F/V intake at month 6 but not at month 12 or 24 compared to UC (Fig. 3A). Other races in the ILI group increased F/V intake at months 6 and 24 but not at month 12 compared to UC (Fig. 3B). Both AA and other race groups in the ILI decreased percent fat at months 6, 12, and 24 compared to UC (Fig. 3C and 3D).

**Fig. 4:**
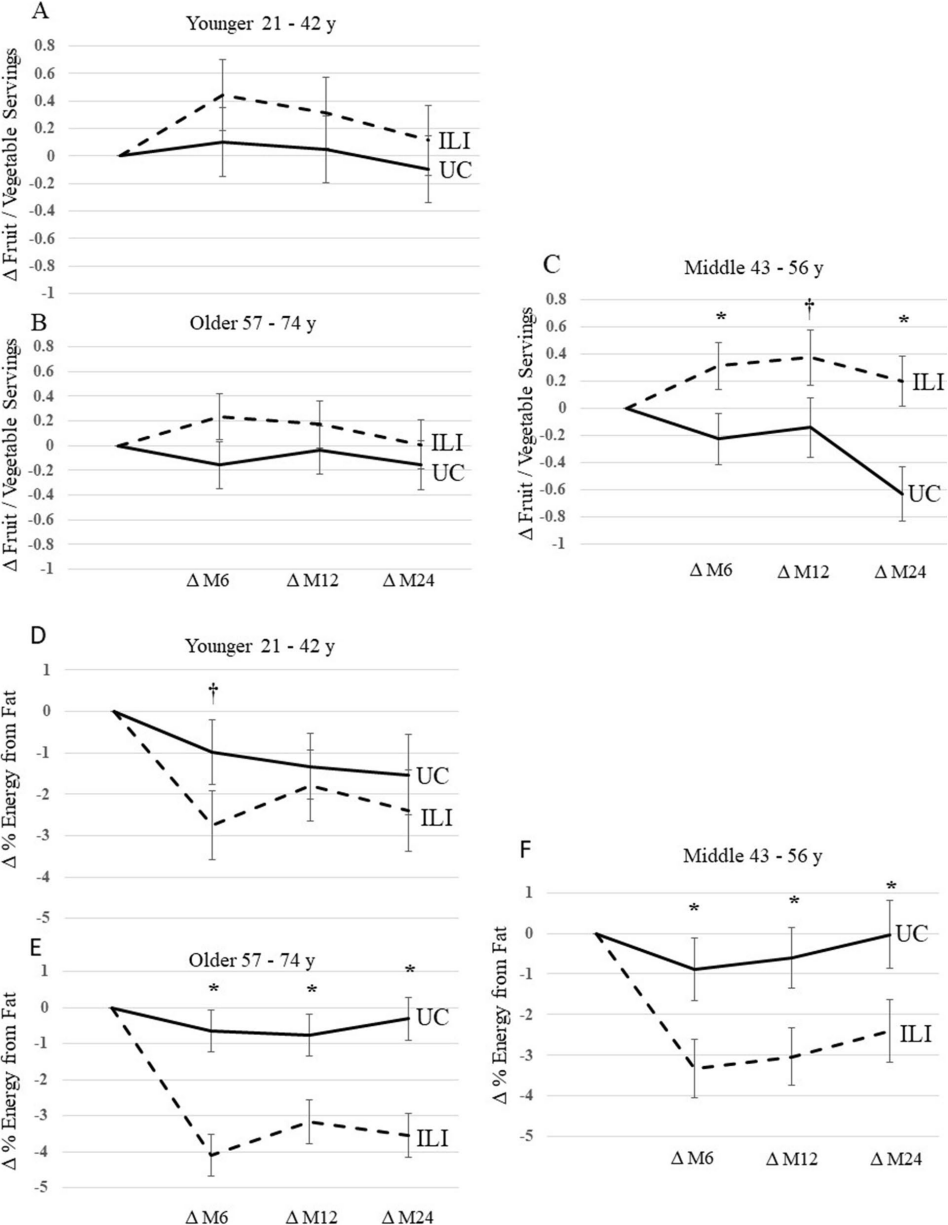
Change in Fruit / Vegetable Intake and % Energy from Fat by Age during the PROPEL trial. 4A and 4D are change in fruit / vegetable servings and percent fat intake in younger patients during the PROPEL trial. 4B and 4E is change in fruit / vegetable servings and percent fat intake in older patients during the PROPEL trial. 4C and 4F are change in fruit / vegetable servings and percent fat intake in middle aged patients during the PROPEL trial. * denotes *p *< 0.05, † denote *p* < 0.07

Age was divided into 3 groups: younger, 21–42 y; middle, 43–56 y; and older, 57–74 y. Younger and older groups in the ILI did not increase F/V servings at months 6, 12, or 24 compared to UC (Fig. 4A and 4B). However, the middle-aged patients in the ILI increased F/V intake at month 6 and 24 compared to UC and tended to increase F/V intake compared to UC at month 12 (Fig. 4C). Younger patients in the ILI did not decrease from fat compared to usual care (Fig. 4D). However, younger patients in ILI tended to reduce percent fat at month 6 compared to UC. Both middle and older patients in the ILI decreased at months 6, 12, and 24 compared to the UC group (Fig. 4E and 4F).

**Fig. 5:**
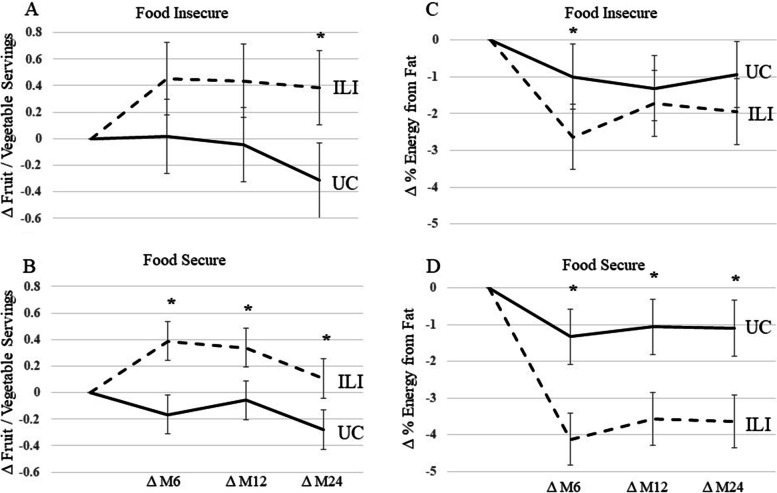
Change in Fruit / Vegetable Intake and % Energy from Fat by Food Security Status during the PROPEL trial. 2A and 2C are change in fruit / vegetable servings and percent fat intake in patients with food security during the PROPEL trial. 2B and 2D are change in fruit / vegetable servings and percent fat intake in patients with food insecurity during the PROPEL trial. * denotes *p* < 0.05

Lastly, the association of food security status and changes in diet were examined. Patients with food insecurity in the ILI increased F/V intake at month 24, but not months 6 or 12, compared to UC (Fig. 5A). Patients with food security in the ILI increased F/V intake at months 6, 12, and 24 compared to UC (Fig. 5B). Patients with food insecurity in the ILI group reduced percent fat at month 6 compared to UC, but not at month 12 or 24 (Fig. 5C). Patients with food security in the ILI reduced percent fat at months 6, 12, and 24 compared to UC (Fig. 5D).

### Analysis for association between intake and weight loss among ILI group

Change in F/V intake was negatively correlated with weight change at month 6 (i.e., increased F/V intake was correlated with more weight loss; Table 1) whereas change in percent energy from fat was positively associated with weight change at months 6, 12, and 24 (i.e., decreased fat intake was correlated with more weight loss; Table 2).

**Table 1:** Results from mixed effects regression models for mean change in body weight for a one unit increase in fruit and vegetable intake among the ILI group, overall and stratified by sex, race, age, and food security status during the 24-month trial

Group	Time	Sample Size	Estimate	95% CI	*P*-Value
ILI	∆ M6	378	-0.5860	[-0.949, -0.224]	**0.0016**
ILI	∆ M12	357	-0.3577	[-0.797, 0.081]	0.1098
ILI	∆ M24	347	-0.1489	[-0.608, 0.310]	0.5237
Sex					
Men	∆ M6	46	-0.4517	[-1.691, 0.788]	0.4646
Men	∆ M12	42	-0.3288	[-1.777, 1.119]	0.6468
Men	∆ M24	40	-0.1459	[-1.927, 1.635]	0.8683
Women	∆ M6	332	-0.6189	[-0.998, -0.24]	**0.0014**
Women	∆ M12	315	-0.3674	[-0.832, 0.097]	0.1204
Women	∆ M24	307	-0.185	[-0.657, 0.287]	0.4411
Race					
AA	∆ M6	278	-0.5897	[-0.967, -0.213]	**0.0023**
AA	∆ M12	262	-0.1989	[-0.634, 0.236]	0.3690
AA	∆ M24	258	-0.0070	[-0.467, 0.453]	0.9760
Other	∆ M6	100	-0.6056	[-1.557, 0.346]	0.2092
Other	∆ M12	95	-0.9925	[-2.247, 0.262]	0.1194
Other	∆ M24	89	-0.6154	[-1.947, 0.716]	0.3604
Age					
Younger	∆ M6	121	-0.3937	[-1.011, 0.224]	0.2090
Younger	∆ M12	105	-0.3425	[-1.085, 0.400]	0.3623
Younger	∆ M24	106	-0.5186	[-1.357, 0.319]	0.2223
Middle	∆ M6	126	-1.0199	[-1.739, -0.301]	**0.0058**
Middle	∆ M12	124	-0.1818	[-0.920, 0.556]	0.6265
Middle	∆ M24	120	-0.4372	[-1.248, 0.374]	0.2876
Older	∆ M6	131	-0.5705	[-1.129, -0.012]	**0.0455**
Older	∆ M12	128	-0.6741	[-1.489, 0.141]	0.1041
Older	∆ M24	121	0.3205	[-0.426, 1.067]	0.3966
Food Security Status					
Secure	∆ M6	272	-0.6067	[-1.039, -0.175]	**0.0061**
Secure	∆ M12	256	-0.3905	[-0.969, 0.188]	0.1847
Secure	∆ M24	249	-0.4617	[-1.052, 0.128]	0.1245
Insecure	∆ M6	106	-0.7629	[-1.413, -0.113]	**0.0219**
Insecure	∆ M12	101	-0.4366	[-1.100, 0.227]	0.1942
Insecure	∆ M24	98	0.1322	[-0.535, 0.800]	0.6948

Sample Size (n). *95% CI* 95% Confidence Interval, *ILI* Intensive lifestyle intervention, *AA* African American. younger, 21–42 y; middle, 43–56 y; and older, 57–74 y

**Table 2:** Results from mixed effects regression models for mean change in body weight for a one percent increase in fat intake among the ILI group, overall and stratified by sex, race, age, and food security status during the 24-month trial

Group	Time	Sample Size	Estimate	95% CI	*P*-Value
ILI	∆ M6	367	0.1647	[0.067, 0.262]	**0.0010**
ILI	∆ M12	349	0.2297	[0.108, 0.351]	**0.0002**
ILI	∆ M24	339	0.2076	[0.086, 0.329]	**0.0009**
Sex					
Men	∆ M6	46	0.2045	[-0.147, 0.556]	0.2462
Men	∆ M12	43	0.1164	[-0.348, 0.581]	0.6136
Men	∆ M24	41	0.2989	[-0.408, 1.005]	0.3947
Women	∆ M6	321	0.1836	[0.083, 0.284]	**0.0004**
Women	∆ M12	306	0.2608	[0.136, 0.386]	** < 0.0001**
Women	∆ M24	298	0.2085	[0.086, 0.331]	**0.0009**
Race					
AA	∆ M6	269	0.1429	[0.034, 0.252]	**0.0103**
AA	∆ M12	255	0.1973	[0.073, 0.322]	**0.0020**
AA	∆ M24	253	0.1259	[-0.004, 0.256]	*0.0581*
Other	∆ M6	98	0.2235	[0.014, 0.433]	**0.0366**
Other	∆ M12	94	0.3306	[0.025, 0.636]	**0.0343**
Other	∆ M24	86	0.3706	[0.085, 0.656]	0.0117
Age					
Younger	∆ M6	116	0.2399	[0.038, 0.442]	**0.0204**
Younger	∆ M12	101	0.3585	[0.118, 0.599]	**0.0039**
Younger	∆ M24	101	0.1900	[-0.028, 0.408]	0.0874
Middle	∆ M6	124	0.1470	[-0.016, 0.310]	*0.0762*
Middle	∆ M12	122	0.1048	[-0.103, 0.313]	0.3202
Middle	∆ M24	120	0.1101	[-0.074, 0.294]	0.2390
Older	∆ M6	127	0.0540	[-0.103, 0.211]	0.4969
Older	∆ M12	126	0.2131	[0.011, 0.416]	**0.0391**
Older	∆ M24	118	0.2827	[0.024, 0.542]	**0.0326**
Food Security Status					
Secure	∆ M6	263	0.1019	[-0.014, 0.218]	0.0845
Secure	∆ M12	250	0.2107	[0.067, 0.354]	**0.0041**
Secure	∆ M24	242	0.1631	[0.022, 0.304]	**0.0233**
Insecure	∆ M6	104	0.2883	[0.110, 0.467]	**0.0018**
Insecure	∆ M12	99	0.2105	[-0.021, 0.442]	*0.0747*
Insecure	∆ M24	97	0.2674	[0.017, 0.518]	**0.0366**

Sample Size (n). *95% CI* 95% Confidence Interval, *ILI* Intensive lifestyle intervention, *AA* African American. younger, 21–42 y; middle, 43–56 y; and older, 57–74 y

Change in F/V intake was not associated with weight change in men. However, they were associated in women at month 6. Change in F/V was associated with weight change in AA at month 6 only. No differences were seen at any time point in other races. Change in F/V intake was not associated with weight change at any time point in younger patients but was in middle-aged and older patients at month 6. Change in F/V intake was associated with weight change at month 6 among patients with food security and food insecurity.

Change in percent fat was associated with weight change in women at months 6, 12, and 24 but not in men. Change in percent fat was associated with weight change at months 6 and 12 in AA and tended to be at month 24. Change in percent fat was associated with weight change at months 6, 12, and 24 in other races. Change in percent fat was not associated with weight change in middle-aged patients although there was a trend at month 6. However, change in percent fat was associated with weight change in younger patients at months 6 and 12 and older patients at months 12 and 24. Change in percent fat was associated with weight change at months 12 and 24 in patients with food security and at months 6 and 24 in patients with food insecurity. Lastly, change in percent fat tended to be associated with weight change at month 12 in patients with food insecurity.

Results for the regressions with diet and waist circumference are shown in Supplemental Tables. Change in F/V intake was not associated with waist circumference overall or by sex or race (Supplemental Table 1). Only older adults demonstrated an association between change in F/V intake and waist circumference, however it was a positive association (i.e., increased F/V intake as correlated with increased waist circumference). Patients with food security change in F/V intake was associated with waist circumference at month 6 in patients with food insecurity.

Change in percent energy from fat was positively associated with waist circumference at months 6, 12, and 24 (i.e., decreased fat intake was correlated with greater waist circumference reductions; Supplemental Table 2). Change in percent fat was associated with change in waist circumference in women at months 6, 12, and 24 but not in men. Change in percent fat was associated with change in waist circumference at months 6 in AA and tended to be at months 12 and 24. Change in percent fat tended to be associated with change in waist circumference at months 6 and 24 in other races. Change in percent fat was associated with change in waist circumference in younger and middle-aged patients at months 6 and older patients at month 24. Lastly, change in percent fat was associated with change in waist circumference at month 6 in patients with food security and at months 6, 12, and 24 in patients with food insecurity.

### Tertile analyses

Lastly, an analysis across tertiles of change in F/V and percent fat was performed (Supplemental Figs. 1, 2, 3 and 4). There was a difference between the three tertiles in change in F/V intake and weight loss at month 6 of ILI (Supplemental Fig. 1). No other differences between tertiles of change in F/V intake and weight loss were seen. There was a difference between the three tertiles of change in fat intake and weight loss at months 6, 12, and 24 (Supplemental Fig. 2). No differences between tertiles of change in F/V intake change and waist circumference were seen (Supplemental Fig. 3). There was a difference between the three tertiles of change in fat intake and waist circumference at months 6, 12, and 24 (Supplemental Fig. 4).

## Discussion

Herein F/V intake was not improved during a lifestyle intervention delivered through primary care in an underserved population. However, percent energy from fat was decreased in the ILI compared to UC at each time point. Furthermore, interesting results were observed in subgroup analyses. Men increased F/V intake throughout the ILI whereas women only increased F/V intake at month 6 compared to UC. However, women decreased percent fat in the ILI vs. UC at all time points, whereas men demonstrated no change in fat intake in ILI vs. UC. Race marginally affected F/V and percent energy from fat intake with differences between groups at month 24 F/V intake in ILI vs. UC. Younger ILI patients did not alter their diet and older ILI participants did not alter their F/V intake compared to UC. Middle-aged ILI patients increased F/V intake at month 6 and 24 in ILI vs. UC and middle-aged and older patients decreased fat intake at all time points compared to UC. Lastly, food secure patients in the ILI were able to increase F/V intake and decrease fat intake at all time points compared to UC. However, food insecure patients in the ILI group were only able to change dietary habits at month 24 for F/V intake and month 6 for fat intake compared to UC.

Changes in percent fat intake in behavioral weight loss interventions have been associated with decreased consumption of high fat foods [27]. However, previous interventions delivered through primary care have not seen differences in percent fat intake [10]. The POWER-UP trial was a similar trial performed in primary care clinics through the University of Pennsylvania Health System [10]. Their baseline F/V servings were over 2.5 × the amount of the current study. On average, their participants exceeded the recommended 4.5 servings of F/V per day at baseline [10]. Likely this reflects the differences in study populations. The baseline percent energy from fat screener was more similar between studies with their patients consuming ~ 33% and PROPEL patients consuming ~ 35% fat intake. Overall, F/V intake targets remained well below recommendations while percent energy from fat was lower following the ILI in PROPEL. Importantly both dietary components (F/V intake and percent energy from fat) are associated with weight loss and should continue to be promoted. However, the partial correlation between F/V intake and weight loss was 0.8 × that of fat intake during the weight loss phase (~ first 6 months). Although, during weight maintenance, F/V intake was not associated with weight change but percent energy from fat remained associated. Thus, while speculative, F/V intake may be more strongly promoted during weight loss whereas decreased fat intake may be promoted during the weight loss and weight maintenance phases.

Limited information is known about the patients from the POWER-UP trial, but they were all from primary care practices within the Philadelphia area associated with the University of Pennsylvania Health System [10, 28]. Out of the six sites, three were urban and three were suburban. Patients enrolled in the trial were 79.7% female, 38.5% AA, and 74.6% had some college or an associate’s degree or higher education level. Whereas in the PROPEL trial, patients came from 14 federally qualified health centers (FQHC’s) from around the state of Louisiana and four clinics were from one large, nonprofit academic subspecialty healthcare delivery system [12]. Also, 14 of the clinics were in urban areas and 4 were in rural areas. Furthermore, as noted herein, participants were 84% female, 56.3% AA, 26.1% receiving Medicaid, 30.8% food insecure, 30.8% had ≤ 8^th^ grade health literacy level, and 65.5% had an income less than $40,000 [11]. Thus, the differences in the populations of the weight loss studies performed in primary care may explain some of the differential findings between the two weight loss studies performed in primary care.

Previously, improvements in dietary quality have been shown to improve weight loss outcomes. However, to our knowledge there is limited evidence this occurs in an underserved population in primary care particularly in various subgroups. Overall, the men’s averages were significant, however most were somewhat modest, particularly with the changes in F/V intake. Men in the ILI nearly doubled the change in F/V intake compared to women. Women in the ILI had more than one percent greater reduction in fat intake than men. Within the ILI group, other races more than doubled the change in F/V intake compared to AA. Other races and AA ILI groups had similar decreases in percent energy from fat during the course of the intervention. With age, middle age was the most responsive to changing diet whereas the younger age group was the least responsive. Younger age had the least dietary changes and the least weight loss in the intervention [11].

The effect of food insecurity on weight loss is not well established [29]. Thus, assessing the relationships between dietary intake and weight loss by food security status is a novel analysis. Previous post-hoc analyses have shown that weight loss was much greater in patients with food security compared to those with food insecurity [29]. Herein, patients with food insecurity in the ILI improved F/V intake, but the high variability (i.e., heterogeneity) in this nutritional outcome prevented significant differences between the ILI and UC groups. Patients with food insecurity failed to dramatically decrease fat intake in response to a weight loss intervention. However, among those that did decrease fat intake during the ILI, associations suggested were more successful with weight loss particularly at months 6 and 24.

Interestingly, one of the most consistent findings across subgroups was that of the association between increased F/V intake and weight loss particularly at the month 6 time point. The association was significant for women, AA, middle aged, older age, food secure, and food insecure subgroups in the ILI. While the associations in men and other races were not significant, the effect was similar as to those above. Only in younger persons was the association somewhat attenuated. Waist circumference is another marker of obesity that provides estimates of central adiposity. While not identical, the main themes between dietary associations with weight loss and waist circumference were similar. Of note was how strong the response remained for the association between waist circumference and fat intake within the overall ILI, in women, and those patients with food insecurity.

The sub-analyses provide some important insights for future interventions. Targeted improvements by sex and age may occur. For example, men could be targeted for greater percent fat intake reductions whereas women could increase F/V intake. Race marginally affected dietary intake, but AA could be targeted for improved F/V consumption. Younger persons likely need more intensive nutritional counseling during lifestyle interventions to improve F/V intake as well as decrease percent energy from fat intake. Lastly, the heterogeneity in response for food insecure patients was quite wide. The average change in F/V intake was fairly robust but the percent energy from fat reduction was blunted in food insecure patients. Future lifestyle interventions may incorporate these results to potentially bolster further weight loss in patients with obesity and food insecurity. While most dietary changes mirrored weight loss, not all dietary responses did. Men and women had similar weight loss but opposing effects on F/V and percent fat intake. Also, AA and all other races similar reduced percent energy from fat in the ILI vs. UC; however, AA weight loss was diminished compared to other races. Lastly, food insecure dietary responses were blunted similar to the body weight data [29].

The U.S. Preventive Services Task Force (USPSTF) has recently published an updated recommendation statement on behavioral counseling intervention to promote healthy diet and physical activity for primary cardiovascular disease prevention in adults [30, 31]. Similar to the ILI performed herein, their healthy diet recommendation is increased consumption of F/V and low-fat or fat-free dairy and lean proteins [30]. Based on our data in the present study, the USPSTF recommendations may be particularly applicable to the underserved AA population [31].

A limitation of the current study is the reliance on dietary screeners. ASA -24 and / or food photography would provide much more granular data including (but not limited to) energy intake, macronutrients, and diet quality. This was a pragmatic trial in an underserved population. Thus overall, the authors feel that the study is generalizable to underserved populations. However, this study was free of charge (complimentary) to patients. Patients received a stipend for attending their clinic visits. Thus, if insurance didn’t pay for the health coaching service patients may not be able to afford to take part in the program. Another potential limitation to this study is the recruitment, training, and compensation of health coaches. However, the study had numerous strengths. The main trial was powered to examine not only differences in body weight but also the planned subgroup analyses of sex, race, and age which are presented herein. Further, the study tested the 2013 AHA/ACC/TOS Obesity Guidelines delivered to a diverse population in primary care through a well-powered clinic randomized controlled trial.

## Conclusion

The ILI decreased percent energy from fat but did not increase F/V intake in a pragmatic trial in primary clinics across Louisiana. Both change in F/V and percent energy from fat were associated with weight loss at month 6, with a decrease in fat intake being associated with weight loss at all time points. On average, F/V intakes were well below guidelines so there is extensive room for improvement in adherence to the dietary guidelines. Future efforts better targeting both increasing F/V intake and reducing fat intake may promote greater weight loss in similar populations. Lastly, the sex, race, age, and food security status sub-analyses provide unique nutritional insights to possibly achieve greater weight loss to potentially target and/or tailor in future weight loss interventions.

## Supplementary Information

**Additional file 1: Supplemental Figure 1.** Mean weight loss in the Intensive Lifestyle Intervention group during the PROPEL trial across low (-1.4 ± 0.1, -1.4 ± 0.1, and -1.8 ± 0.1 servings per day at months 6, 12, and 24, respectively), moderate (0.2 ± 0.0, 0.2 ± 0.0, and 0.1 ± 0.0 servings per day at months 6, 12, and 24, respectively) and high (2.0 ± 0.1, 1.9 ± 0.1, and 1.8 ± 0.1 servings per day at months 6, 12, and 24, respectively) tertiles of change in fruit / vegetable intake. Error bars represent SEM. Letters with a different superscript differ by *p*<0.05.

**Additional file 2: Supplemental Figure 2.** Mean weight loss in the Intensive Lifestyle Intervention group during the PROPEL trial across high (-10.0 ± 0.5, -9.0 ± 0.5, and -9.7 ± 0.6 percent fat at months 6, 12, and 24, respectively), moderate (-2.8 ± 0.1, -2.1 ± 0.1, and -2.1 ± 0.1 percent fat at months 6, 12, and 24, respectively) and low (1.8 ± 0.2, 2.5 ± 0.3, and 2.6 ± 0.4 percent fat at months 6, 12, and 24, respectively) tertiles of change percent energy from fat. Error bars represent SEM. Letters with a different superscript differ by *p*<0.05.

**Additional file 3: Supplemental Figure 3.** Mean waist circumference in the Intensive Lifestyle Intervention group during the PROPEL trial across low (-1.4 ± 0.1, -1.4 ± 0.1, and -1.8 ± 0.1servings per day at months 6, 12, and 24, respectively), moderate (0.2 ± 0.0, 0.2 ± 0.0, and 0.1 ± 0.0 servings per day at months 6, 12, and 24, respectively) and high (2.0 ± 0.1, 1.9 ± 0.1, and 1.8 ± 0.1 servings per day at months 6, 12, and 24, respectively) tertiles of change in fruit / vegetable intake. Error bars represent SEM.

**Additional file 4: Supplemental Figure 4.** Mean waist circumference in the Intensive Lifestyle Intervention group during the PROPEL trial across high (-10.0 ± 0.5, -9.0 ± 0.5, and -9.7 ± 0.6 percent fat at months 6, 12, and 24, respectively), moderate (-2.8 ± 0.1, -2.1 ± 0.1, and -2.1 ± 0.1 percent fat at months 6, 12, and 24, respectively) and low (1.8 ± 0.2, 2.5 ± 0.3, and 2.6 ± 0.4 percent fat at months 6, 12, and 24, respectively) tertiles of change percent energy from fat. Error bars represent SEM. Letters with a different superscript differ by *p*<0.05.

**Additional file 5: Supplemental Table 1.** Results from mixed effects regression models for mean change in waist circumference for a one unit increase in fruit and vegetable intake among the ILI group, overall and stratified by sex, race, age, and food security status during the 24-month trial. **Supplemental Table 2.** Results from mixed effects regression models for mean change in waist circumference for a one percent increase in fat intake among the ILI group, overall and stratified by sex, race, age, and food security status during the 24-month trial.

## Data Availability

De-identified data will be made available upon reasonable request.

## Abbreviations

PROPEL: Promoting Successful Weight Loss in Primary Care in Louisiana
ILI: Intensive Lifestyle Intervention
UC: Usual Care
F/V: Fruits and Vegetables
AA: African American
