# Supplementary figures and images for: Dietary intake during a pragmatic cluster-randomized weight loss trial in an underserved population in primary care
Source: Nutr J. 2023 Aug 2;22:38. doi: 10.1186/s12937-023-00864-7 (PMC10394871; doi:10.1186/s12937-023-00864-7)

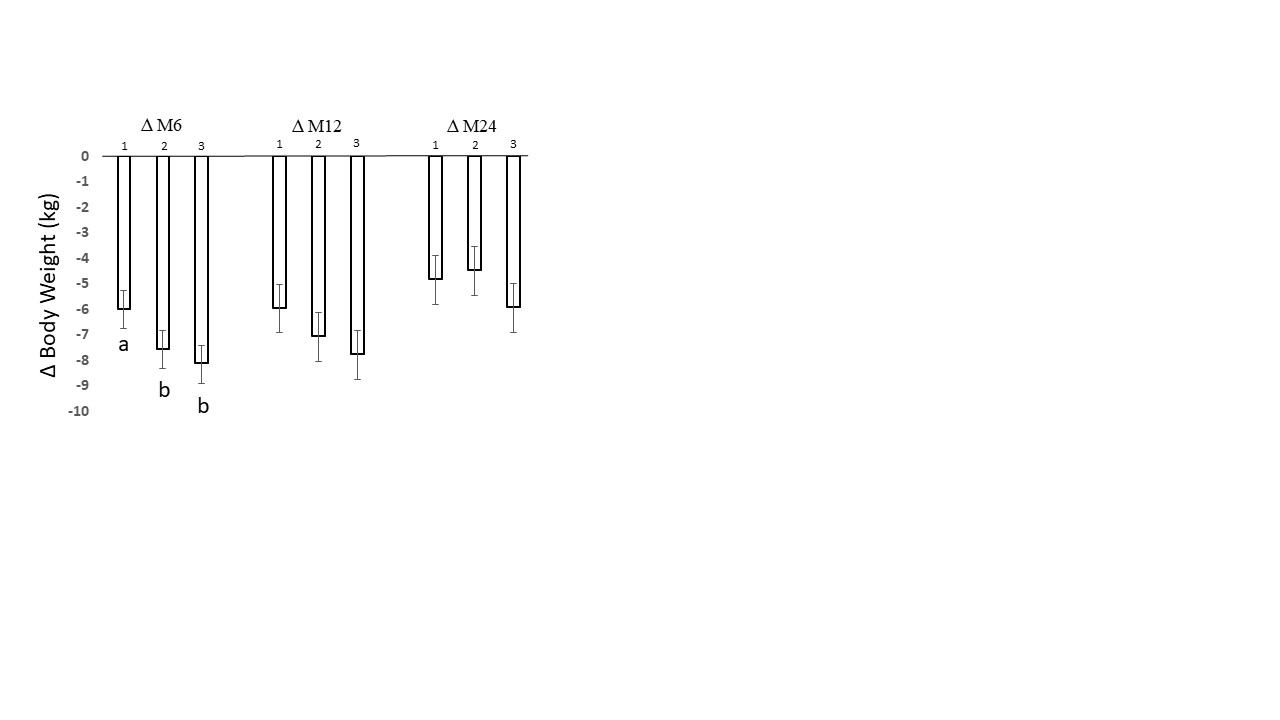

Supplement: Supplementary file 1 — Additional file 1: Supplemental Figure 1. Mean weight loss in the Intensive Lifestyle Intervention group during the PROPEL trial across low (-1.4 ± 0.1, -1.4 ± 0.1, and -1.8 ± 0.1 servings per day at months 6, 12, and 24, respectively), moderate (0.2 ± 0.0, 0.2 ± 0.0, and 0.1 ± 0.0 servings per day at months 6, 12, and 24, respectively) and high (2.0 ± 0.1, 1.9 ± 0.1, and 1.8 ± 0.1 servings per day at months 6, 12, and 24, respectively) tertiles of change in fruit / vegetable intake. Error bars represent SEM. Letters with a different superscript differ by p<0.05. [file 12937_2023_864_MOESM1_ESM.jpg]

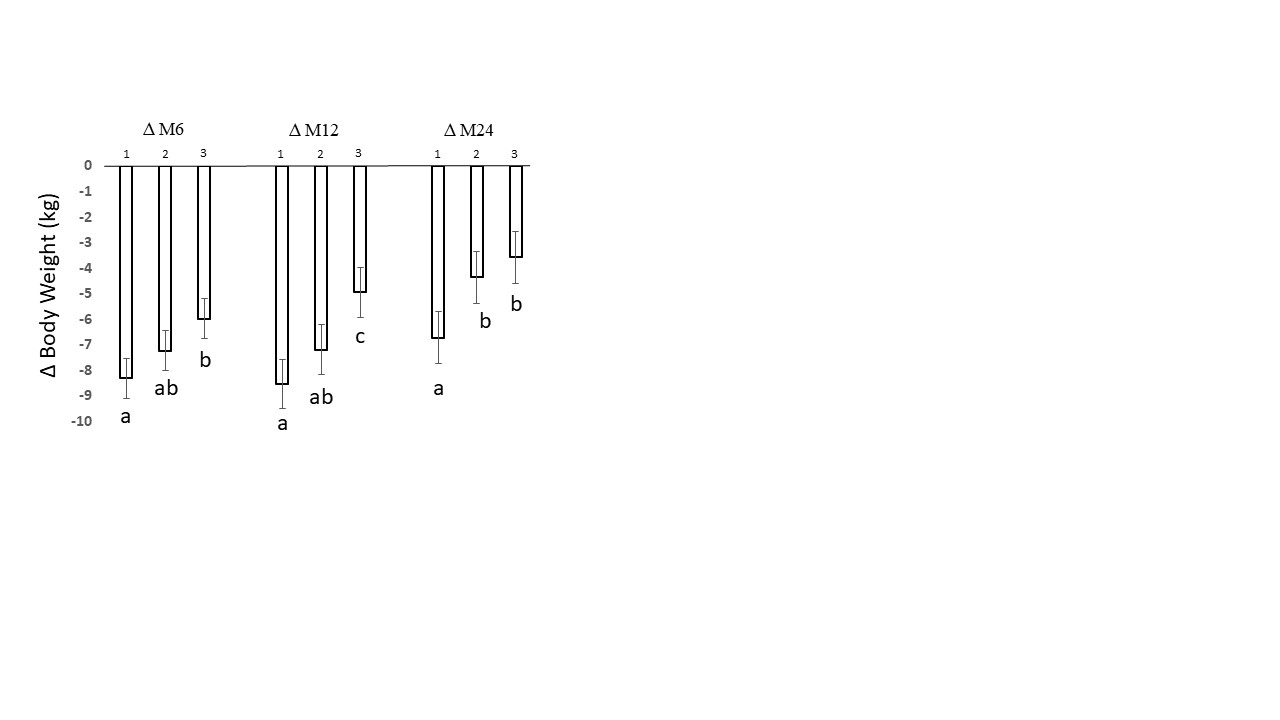

Supplement: Supplementary file 2 — Additional file 2: Supplemental Figure 2. Mean weight loss in the Intensive Lifestyle Intervention group during the PROPEL trial across high (-10.0 ± 0.5, -9.0 ± 0.5, and -9.7 ± 0.6 percent fat at months 6, 12, and 24, respectively), moderate (-2.8 ± 0.1, -2.1 ± 0.1, and -2.1 ± 0.1 percent fat at months 6, 12, and 24, respectively) and low (1.8 ± 0.2, 2.5 ± 0.3, and 2.6 ± 0.4 percent fat at months 6, 12, and 24, respectively) tertiles of change percent energy from fat. Error bars represent SEM. Letters with a different superscript differ by p<0.05. [file 12937_2023_864_MOESM2_ESM.jpg]

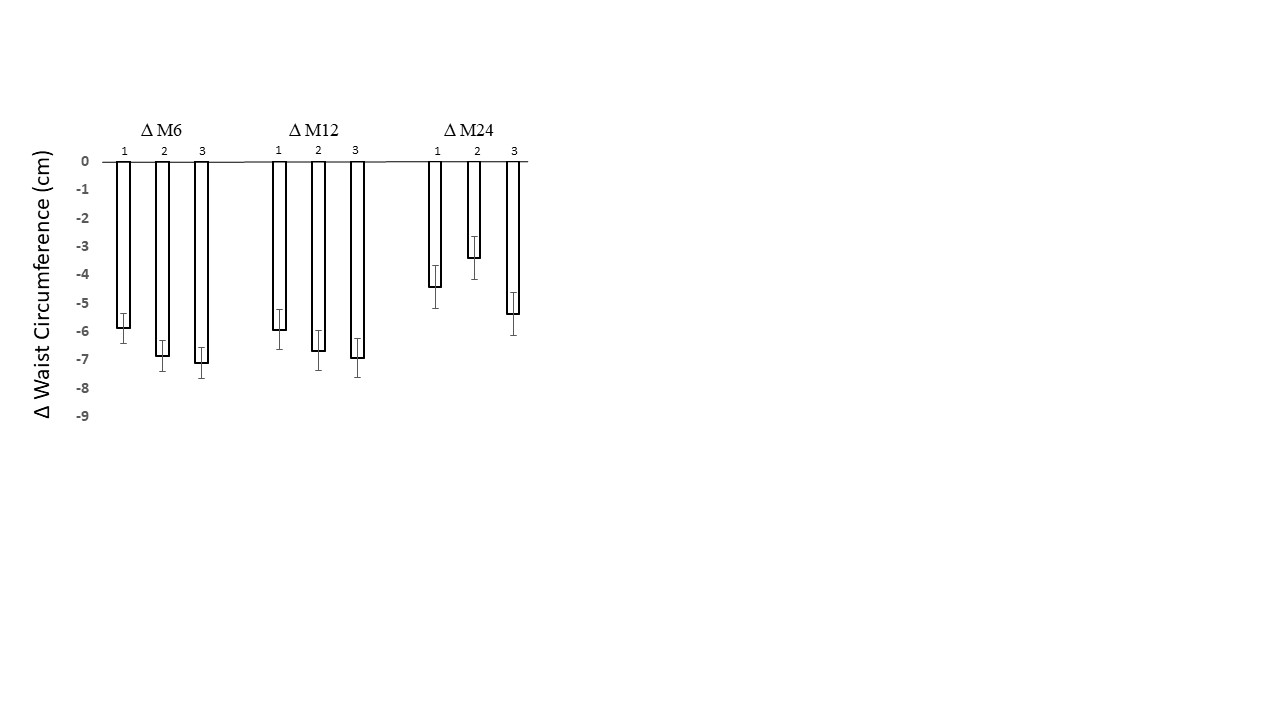

Supplement: Supplementary file 3 — Additional file 3: Supplemental Figure 3. Mean waist circumference in the Intensive Lifestyle Intervention group during the PROPEL trial across low (-1.4 ± 0.1, -1.4 ± 0.1, and -1.8 ± 0.1servings per day at months 6, 12, and 24, respectively), moderate (0.2 ± 0.0, 0.2 ± 0.0, and 0.1 ± 0.0 servings per day at months 6, 12, and 24, respectively) and high (2.0 ± 0.1, 1.9 ± 0.1, and 1.8 ± 0.1 servings per day at months 6, 12, and 24, respectively) tertiles of change in fruit / vegetable intake. Error bars represent SEM. [file 12937_2023_864_MOESM3_ESM.jpg]

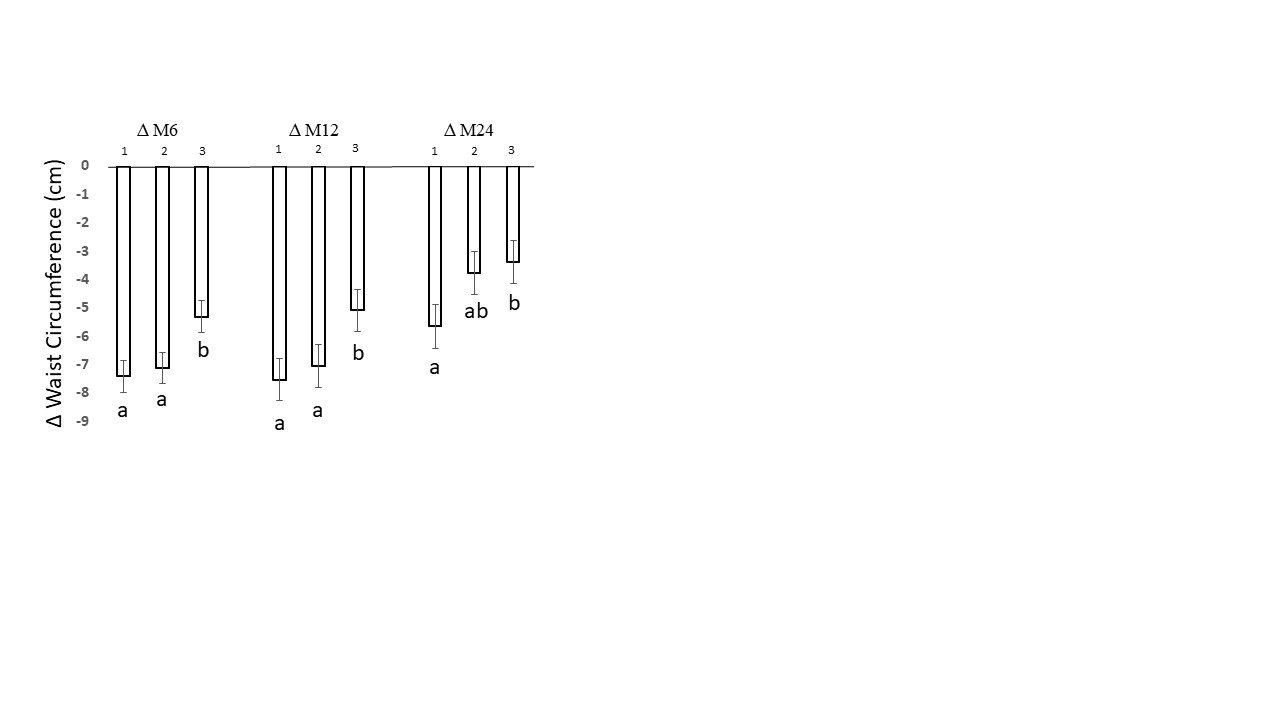

Supplement: Supplementary file 4 — Additional file 4: Supplemental Figure 4. Mean waist circumference in the Intensive Lifestyle Intervention group during the PROPEL trial across high (-10.0 ± 0.5, -9.0 ± 0.5, and -9.7 ± 0.6 percent fat at months 6, 12, and 24, respectively), moderate (-2.8 ± 0.1, -2.1 ± 0.1, and -2.1 ± 0.1 percent fat at months 6, 12, and 24, respectively) and low (1.8 ± 0.2, 2.5 ± 0.3, and 2.6 ± 0.4 percent fat at months 6, 12, and 24, respectively) tertiles of change percent energy from fat. Error bars represent SEM. Letters with a different superscript differ by p<0.05. [file 12937_2023_864_MOESM4_ESM.jpg]
