# Supplementary material for: Dietary intake during a pragmatic cluster-randomized weight loss trial in an underserved population in primary care
Source: Nutr J. 2023 Aug 2;22:38. doi: 10.1186/s12937-023-00864-7 (PMC10394871; doi:10.1186/s12937-023-00864-7)
Supplement: Supplementary file 5 — Additional file 5: Supplemental Table 1. Results from mixed effects regression models for mean change in waist circumference for a one unit increase in fruit and vegetable intake among the ILI group, overall and stratified by sex, race, age, and food security status during the 24-month trial. Supplemental Table 2. Results from mixed effects regression models for mean change in waist circumference for a one percent increase in fat intake among the ILI group, overall and stratified by sex, race, age, and food security status during the 24-month trial. [file 12937_2023_864_MOESM5_ESM.docx]

Supplemental Table 1: Results from mixed effects regression models for mean change in waist circumference for a one unit increase in fruit and vegetable intake among the ILI group, overall and stratified by sex, race, age, and food security status during the 24-month trial

Group Time Sample Estimate 95% CI P-Value

Size

ILI ∆ M6 377 -0.3417 [-0.715, 0.032] *0.0728*

ILI ∆ M12 356 -0.1241 [-0.561, 0.312] 0.5762

ILI ∆ M24 346 0.01413 [-0.421, 0.450] 0.9491

Sex

Men ∆ M6 46 -0.1467 [-1.241, 0.948] 0.7872

Men ∆ M12 42 0.2974 [-1.066, 1.661] 0.6597

Men ∆ M24 40 -0.1233 [-1.686, 1.439] 0.8730

Women ∆ M6 331 -0.3187 [-0.715, 0.078] 0.1145

Women ∆ M12 314 -0.1688 [-0.631, 0.294] 0.4732

Women ∆ M24 306 0.03292 [-0.422, 0.488] 0.8869

Race

AA ∆ M6 277 -0.1929 [-0.598, 0.213] 0.3498

AA ∆ M12 261 0.09308 [-0.384, 0.570] 0.7010

AA ∆ M24 257 0.2923 [-0.168, 0.752] 0.2121

Other ∆ M6 100 -0.6596 [-1.549, 0.230] 0.1442

Other ∆ M12 95 -0.7097 [-1.712, 0.293] 0.1628

Other ∆ M24 89 -0.8865 [-2.028, 0.255] 0.1261

Age

Younger ∆ M6 121 -0.2022 [-0.817, 0.413] 0.5162

Younger ∆ M12 105 -0.1996 [-0.916, 0.516] 0.5811

Younger ∆ M24 106 -0.3652 [-1.124, 0.394] 0.3420

Middle ∆ M6 125 -0.7146 [-1.458, 0.029] *0.0594*

Middle ∆ M12 123 -0.0395 [-0.754, 0.675] 0.9131

Middle ∆ M24 119 -0.6219 [-1.344, 0.101] 0.0908

Older ∆ M6 131 -0.2025 [-0.801, 0.396] 0.5039

Older ∆ M12 128 -0.2344 [-1.085, 0.616] 0.5862

Older ∆ M24 121 0.7912 [0.036, 1.546] **0.0401**

Food Security Status

Secure ∆ M6 271 -0.5089 [-0.96, -0.058] **0.0271**

Secure ∆ M12 255 -0.3545 [-0.933, 0.224] 0.2289

Secure ∆ M24 248 -0.3981 [-0.950, 0.154] 0.1566

Insecure ∆ M6 106 -0.0336 [-0.692, 0.625] 0.9196

Insecure ∆ M12 101 0.0566 [-0.590, 0.703] 0.8625

Insecure ∆ M24 98 0.6808 [-0.001, 1.363] *0.0503*

Sample Size (n). 95% CI, 95% Confidence Interval. ILI, intensive lifestyle intervention. AA, African American. younger, 21-42 y; middle, 43-56 y; and older, 57-74 y.

Supplemental Table 2: Results from mixed effects regression models for mean change in waist circumference for a one percent increase in fat intake among the ILI group, overall and stratified by sex, race, age, and food security status during the 24-month trial

Group Time Sample Estimate 95% CI P-Value

Size

ILI ∆ M6 366 0.1647 [0.076, 0.277] **0.0010**

ILI ∆ M12 348 0.2297 [0.042, 0.287] **0.0002**

ILI ∆ M24 338 0.2076 [0.056, 0.291] **0.0009**

Sex

Men ∆ M6 46 0.1655 [-0.125, 0.456] 0.2551

Men ∆ M12 43 0.1339 [-0.297, 0.565] 0.5316

Men ∆ M24 41 0.1757 [-0.431, 0.783] 0.5592

Women ∆ M6 320 0.200 [0.094, 0.306] **0.0002**

Women ∆ M12 305 0.181 [0.053, 0.309] **0.0057**

Women ∆ M24 297 0.1772 [0.057, 0.297] **0.0039**

Race

AA ∆ M6 268 0.1731 [0.058, 0.288] **0.0033**

AA ∆ M12 254 0.1375 [-0.001, 0.276] *0.0516*

AA ∆ M24 252 0.1413 [0.009, 0.274] **0.0365**

Other ∆ M6 98 0.1807 [-0.020, 0.381] *0.0767*

Other ∆ M12 94 0.2223 [-0.031, 0.476] 0.0846

Other ∆ M24 86 0.2385 [-0.011, 0.488] *0.061*

Age

Younger ∆ M6 116 0.2301 [0.033, 0.428] **0.0228**

Younger ∆ M12 101 0.2026 [-0.030, 0.435] 0.0863

Younger ∆ M24 101 0.1494 [-0.043, 0.342] 0.1268

Middle ∆ M6 123 0.1664 [0.004, 0.329] **0.0447**

Middle ∆ M12 121 0.1464 [-0.051, 0.344] 0.144

Middle ∆ M24 119 0.05216 [-0.119, 0.224] 0.5477

Older ∆ M6 127 0.08828 [-0.083, 0.260] 0.3095

Older ∆ M12 126 0.142 [-0.077, 0.361] 0.2015

Older ∆ M24 118 0.3651 [0.104, 0.627] **0.0066**

Food Security Status

Secure ∆ M6 262 0.1513 [0.032, 0.271] **0.0132**

Secure ∆ M12 249 0.1179 [-0.029, 0.264] 0.1142

Secure ∆ M24 241 0.1267 [-0.005, 0.259] *0.0601*

Insecure ∆ M6 104 0.2329 [0.045, 0.421] **0.0155**

Insecure ∆ M12 99 0.2611 [0.034, 0.488] **0.0245**

Insecure ∆ M24 97 0.3253 [0.063, 0.587] **0.0155**

Sample Size (n). 95% CI, 95% Confidence Interval. ILI, intensive lifestyle intervention. AA, African American. younger, 21-42 y; middle, 43-56 y; and older, 57-74 y.
